# Progression-free survival at 24 months and subsequent survival of patients with extranodal NK/T-cell lymphoma: a China Lymphoma Collaborative Group (CLCG) study

**DOI:** 10.1038/s41375-020-01042-y

**Published:** 2020-09-17

**Authors:** Yong Yang, Ying Wang, Xin Liu, Xia He, Li-Ling Zhang, Gang Wu, Bao-Lin Qu, Li-Ting Qian, Xiao-Rong Hou, Fu-Quan Zhang, Xue-Ying Qiao, Hua Wang, Gao-Feng Li, Yuan Zhu, Jian-Zhong Cao, Jun-Xin Wu, Tao Wu, Su-Yu Zhu, Mei Shi, Li-Ming Xu, Hang Su, Yu-Qin Song, Jun Zhu, Yu-Jing Zhang, Hui-Qiang Huang, Chen Hu, Shu-Nan Qi, Ye-Xiong Li

**Affiliations:** 1 State Key Laboratory of Molecular Oncology and Department of Radiation Oncology, National Cancer Center/Cancer Hospital, Chinese Academy of Medical Sciences (CAMS) and Peking Union Medical College (PUMC), Beijing, China; 2 Chongqing University Cancer Hospital & Chongqing Cancer Hospital, Chongqing, China; 3 Jiangsu Cancer Hospital & Jiangsu Institute of Cancer Research, Nanjing, Jiangsu China; 4 Union Hospital, Tongji Medical College, Huazhong University of Science and Technology, Wuhan, Hubei China; 5 The General Hospital of Chinese People’s Liberation Army, Beijing, China; 6 The Affiliated Provincial Hospital of Anhui Medical University, Hefei, Anhui China; 7 Peking Union Medical College Hospital, Chinese Academy of Medical Sciences (CAMS) and Peking Union Medical College (PUMC), Beijing, China; 8 The Fourth Hospital of Hebei Medical University, Shijiazhuang, China; 9 Second Affiliated Hospital of Nanchang University, Nanchang, China; 10 Beijing Hospital, National Geriatric Medical Center, Beijing, China; 11 Cancer Hospital of the University of Chinese Academy of Sciences, Zhejiang Cancer Hospital, Hangzhou, Zhejiang China; 12 Shanxi Cancer Hospital and the Affiliated Cancer Hospital of Shanxi Medical University, Taiyuan, Shanxi China; 13 Fujian Provincial Cancer Hospital, Fuzhou, Fujian China; 14 Affiliated Hospital of Guizhou Medical University, Guizhou Cancer Hospital, Guiyang, Guizhou China; 15 Hunan Cancer Hospital and the Affiliated Cancer Hospital of Xiangya School of Medicine, Changsha, Hunan China; 16 Xijing Hospital of Fourth Military Medical University, Xi’an, China; 17 Tianjin Medical University Cancer Institute & Hospital, Key Laboratory of Cancer Prevention and Therapy, National Clinical Research Center for Cancer, Tianjin, China; 18 The Fifth Medical Center of PLA General Hospital, Beijing, China; 19 Key Laboratory of Carcinogenesis and Translational Research (Ministry of Education), Peking University Cancer Hospital & Institute, Beijing, China; 20 Sun Yat-Sen University Cancer Center; State Key Laboratory of Oncology in South China; Collaborative Innovation Center for Cancer Medicine, Guangzhou, Guangdong China; 21 Division of Biostatistics and Bioinformatics, Sidney Kimmel Comprehensive Cancer Center, Johns Hopkins University School of Medicine, Baltimore, MD 21205-2013 USA

**Keywords:** T-cell lymphoma, Risk factors

## Abstract

Limited evidence supports the use of early endpoints to evaluate the success of initial treatment of extranodal NK/T-cell lymphoma (ENKTCL) in the modern era. We aim to analyze progression-free survival at 24 months (PFS24) and subsequent overall survival (OS) in a large-scale multicenter cohort of patients. 1790 patients were included from the China Lymphoma Collaborative Group (CLCG) database. Subsequent OS was defined from the time of PFS24 or progression within 24 months to death. OS was compared with age- and sex-matched general Chinese population using expected survival and standardized mortality ratio (SMR). Patients who did not achieve PFS24 had a median OS of 5.3 months after progression, with 5-year OS rate of 19.2% and the SMR of 71.4 (95% CI, 62.9–81.1). In contrast, 74% patients achieved PFS24, and the SMR after achieving PFS24 was 1.77 (95% CI, 1.34–2.34). The observed OS rate after PFS24 versus expected OS rate at 5 years was 92.2% versus 94.3%. Similarly, superior outcomes following PFS24 were observed in early-stage patients (5-year OS rate, 92.9%). Patients achieving PFS24 had excellent outcome, whereas patients exhibiting earlier progression had a poor survival. These marked differences suggest that PFS24 may be used for study design and risk stratification in ENKTCL.

## Introduction

Extranodal NK/T-cell lymphoma, nasal type (ENKTCL) is an aggressive and heterogeneous disease with an unusual geographical distribution. Although rare globally, it is more prevalent in East Asia and South America [1–4]. Clinically, the majority of patients with ENKTCL present with early-stage disease and are male adults. Extensive primary tumor invasion (PTI) is usually present, involving the upper aerodigestive tract (UADT) site, and extranodal failure is common [5–7]. Over the past decade, substantial treatment advances have been made with the introduction of novel prognostic models and risk stratifications [8–10], upfront radiotherapy [11–14], and non-anthracycline (ANT)-based chemotherapy [15–21]. The treatment outcome has improved, with 5-year overall survival (OS) rates of 60–90% reported for localized diseases, but only 10–40% for disseminated diseases [10, 13, 22–25]. A large proportion of patients suffers disease progression or relapse after employment of the current standard treatment. Thus, there is an urgent requirement for the identification of early efficacy endpoints or novel therapeutic agents from prospective trials in patients with ENKTCL.

Previous studies have demonstrated that event-free survival (EFS) at 24 months (EFS24) and progression-free survival (PFS) at 24 months (PFS24) are important milestones to stratify patients with diffuse large B-cell lymphoma (DLBCL) and peripheral T-cell lymphoma (PTCL); survival beyond these time points is similar or equivalent to that of the general population [26–29]. Normalization to the survival of a country-matched general population was recently proposed as an alternative to the use of “time to event” analysis for lymphoma. However, clinical characteristics and treatment strategies differ notably between lymphoma subtypes. The clinical significance of PFS24 in ENKTCL remains unknown, and the impact of achieving PFS24 on subsequent OS remains unaddressed. In the present study, we aimed to examine the timing of events, post-treatment milestones and subsequent OS in patients with ENKTCL, with reference to the general background population.

## Patients and methods

### Eligibility criteria and study population

Patients diagnosed with ENKTCL between 2008 and 2016 were retrospectively reviewed using information from the China Lymphoma Collaborative Group (CLCG) database [5, 9, 13, 21]. The eligibility criteria in this study included patients who had received non-ANT-based chemotherapy and/or radiotherapy. Patients who had received ANT-based or unknown regimen chemotherapy were excluded. A total of 1790 patients formed the study population. The institutional review boards ethically approved this project and waived the requirement for informed consent because of the de-identification of patient data.

### Risk stratification and treatment

Patients were staged using the Ann Arbor staging system and stratified using the ENKTCL-specific models: the nomogram-revised risk index [8, 9], the prognostic index of natural killer lymphoma [10] and the Korea prognostic index [30]. Patients with early-stage disease received combined modality treatment (*n* = 1091, 69.8%), radiotherapy alone (*n* = 283, 18.1%) or chemotherapy alone (*n* = 189, 12.1%). Patients with advanced-stage disease received primary chemotherapy with (*n* = 89, 39.2%) or without (*n* = 138, 60.8%) consolidation radiotherapy. The most commonly used non-ANT-based chemotherapies were asparaginase- or platinum-containing (*n* = 1367, 90.7%). Extended involved-site radiation therapy (E-ISRT) was administered, with a median dose of 50 Gy.

### Statistical methods

Progression-free survival (PFS) was defined as the time from the date of treatment to the first of either disease progression, relapse or death from any cause. Subsequent OS was defined as the time from achieving PFS24 (24 months after treatment) or time from progression in patients who did not achieve PFS24 (progression within 24 months of treatment) to death from any cause. The estimated hazard rates provide the trajectory of progression and death overtime and were smoothed by the Epanechnikov kernel. PFS24 was defined as being alive and progression-free 24 months after initial therapy. PFS was also evaluated at other landmark time points (12 and 36 months) for sensitivity analysis. OS was compared with the age- and sex-matched general Chinese population via standardized mortality ratios (SMRs), and expected survival was predicted using a conditional approach via the ‘survexp’ function in R (package survival). Survival curves we analyzed using Kaplan–Meier method. A two-sided *P* value of < 0.05 was considered to indicate a statistically significant difference. Statistical analyses were performed using SPSS (version 22.0; IBM Inc.) and R (version 3.6.2; http://www.r-project.org/).

## Results

### Patient characteristics and survival

The baseline clinical characteristics of the included patients are listed in Table 1. The median age was 44 years old (interquartile range, 32–55 years) and the male:female ratio was 2.4:1. Most patients had good performance status and primary UADT site. Elevated lactate dehydrogenase was noted in 27.6% of patients, and the majority exhibited early-stage disease (87.3%).

**Table 1:** Clinical characteristics and treatment outcomes of patients with extranodal nasal-type NK/T-cell lymphoma.

	Patients	5-year OS rate post-treatment		SMR	
Characteristics	No. (%)	% (95% CI)	*P*	(95% CI)	*P*^a^
Sex			0.181		
Male	1261 (70.4)	71.7 (68.9–74.6)		5.6 (5.1–6.3)	<0.001
Female	529 (29.6)	74.5 (70.4–78.7)		1.3 (1.1–1.5)	<0.001
Age (years)			0.013		
≤60	1527 (85.3)	73.5 (71.1–76.0)		14.7 (13.2–16.3)	<0.001
>60	263 (14.7)	66.4 (59.9–73.6)		1.9 (1.5–2.3)	<0.001
Primary site			<0.001		
UADT	716 (40.0)	73.9 (71.6–76.3)		6.3 (5.7–7.0)	<0.001
Extra-UADT	1074 (60.0)	45.4 (34.0–60.8)		14.3 (10.6–19.3)	<0.001
Regional lymph nodes			<0.001		
Yes	1674 (93.5)	64.7 (60.9–68.8)		13.3 (11.6–15.2)	<0.001
No	116 (6.5)	77.4 (74.6–80.3)		4.5 (3.9––5.1)	<0.001
Distant lymph nodes			<0.001		
Yes	1671 (93.4)	46.3 (35.7–60.2)		17.8 (13.2–24.2)	<0.001
No	119 (5.6)	73.8 (71.5–76.2)		6.2 (5.7–6.9)	<0.001
Primary tumor invasion			<0.001		
Yes	1004 (56.1)	67.9 (64.8–71.2)		9.7 (8.6–10.8)	<0.001
No	786 (43.9)	78.4 (75.1–81.8)		4.1 (3.5–4.8)	<0.001
B symptoms			0.006		
Yes	687 (38.4)	68.9 (65.2–72.9)		9.9 (8.6–11.5)	<0.001
No	1103 (61.6)	74.8 (71.9–77.8)		5.3 (4.6–6.0)	<0.001
Elevated LDH			<0.001		
Yes	100 (5.6)	60.1 (55.3–65.3)		1.2 (1.1–1.4)	<0.001
No	1690 (94.4)	77.1 (74.5–79.7)		5.1 (4.5–5.8)	<0.001
ECOG score			<0.001		
0–1	1563 (87.3)	74.7 (72.4–77.1)		6.2 (5.6–6.8)	<0.001
≥2	227 (12.7)	39.1 (30.2–50.7)		12.9 (10.1–16.5)	<0.001
Ann Arbor stage			<0.001		
I-II	1296 (72.4)	75.9 (73.5–78.3)		5.5 (5.0–6.1)	<0.001
III-IV	494 (27.6)	44.4 (36.7–53.7)		24.4 (19.9–29.8)	<0.001
KPI			<0.001		
Group 1	550 (30.7)	81.8 (78.3–85.5)		3.1 (2.5–3.8)	<0.001
Group 2	636 (35.5)	75.0 (71.2–79.0)		6.2 (5.2–7.3)	<0.001
Group 3	373 (20.9)	67.4 (62.4–72.9)		10.6 (8.7–12.8)	<0.001
Group 4	231 (12.9)	49.6 (42.6–57.9)		27.9 (22.9–34.1)	<0.001
PINK			<0.001		
Low risk	1269 (70.9)	78.1 (75.6–80.6)		11.4 (10.1–12.9)	<0.001
Intermediate risk	363 (20.3)	61.7 (55.9–68.1)		3.1 (2.6–3.7)	<0.001
High risk	158 (8.8)	45.5 (36.6–56.6)		12.8 (10.0–16.3)	<0.001
NRI			<0.001		
Low risk	390 (21.8)	86.4 (82.6–90.3)		5.6 (4.2–7.4)	<0.001
Intermediate-low risk	517 (28.9)	78.3 (74.3–82.5)		3.9 (3.2–4.8)	<0.001
Intermediate-high risk	469 (26.2)	69.8 (65.3–74.8)		7.0 (5.9–8.4)	<0.001
High risk	263 (14.7)	59.4 (52.9–66.6)		11.0 (8.9–13.6)	<0.001
Very high risk	151 (8.4)	43.2 (24.8–53.7)		12.2 (9.7–15.3)	<0.001

*OS* overall survival, *SMR* standardized mortality ratio, *CI* confidence interval, *UADT* upper aerodigestive tract, *LDH* lactate dehydrogenase, *ECOG* Eastern Cooperative Oncology Group, *KPI* Korean Prognostic Index, *PINK* Prognostic Index of Natural Killer Lymphoma, *NRI* nomogram-revised risk index.

^a^Compared with the age- and sex-matched general Chinese population.

With a median follow-up time of 46 months for surviving patients, 598 patients (33.4%) exhibited disease progression and 430 patients (24.0%) passed away. The 5-year OS and PFS rates for all patients were 72.5% and 62.2%, respectively.

### Annual hazard rate over time

**Fig. 1:**
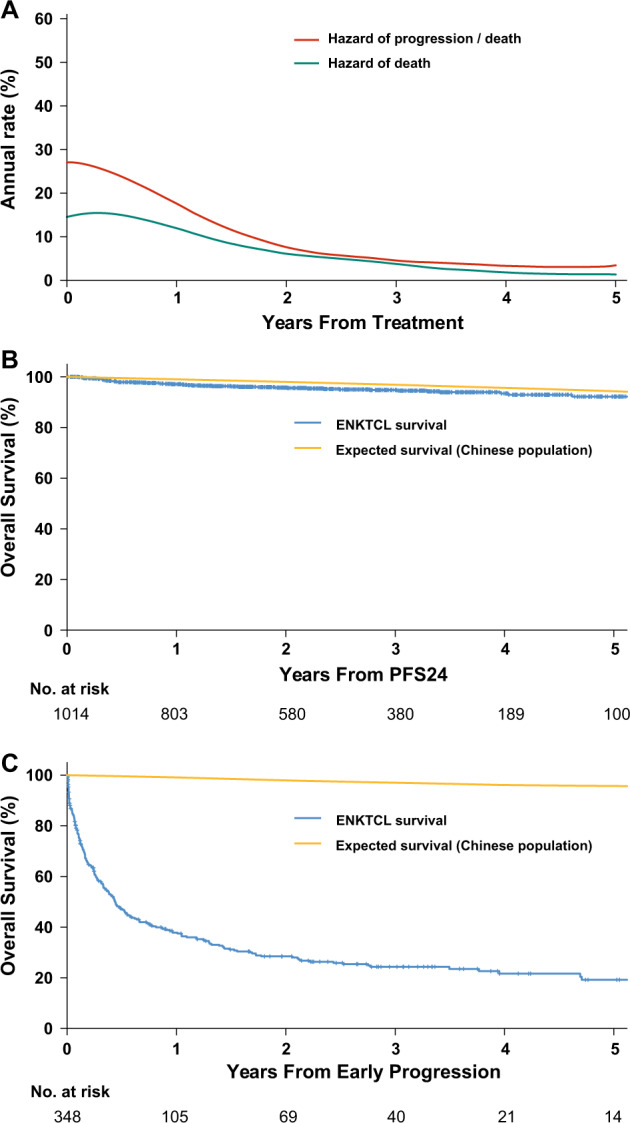
Estimated annual hazards and overall survival (OS) based on 24-month progression-free survival (PFS24) in the whole cohort. **a** Smoothed hazard plots of death and progression over time. **b** Subsequent OS of patients who achieved PFS24 after initial treatment vs. expected OS based on age-, sex-, and country-matched general population data. **c** Subsequent OS of patients who did not achieve PFS24 after initial treatment vs. expected OS based on age-, sex-, and country-matched general population data.

After initial treatment, 82% of progression and 80% of mortalities occurred within 24 months. Consistently, smoothed hazard plots illustrated maximal annual death and progression hazards of 15.4% and 27.1%, respectively during the first year. These hazard rates decreased to 6.1% and 7.6%, respectively, at 2 years (Fig. 1a). From 3 years the annual hazards for progression and death were <5%. Thus, PFS at 24 months (PFS24) is a logistical cutoff time point for further evaluation.

### PFS24 and subsequent OS

Kaplan–Meier analysis estimated that the 2-year PFS rate of the total cohort (1790 patients) was 70.5% (95% CI, 68.3–72.7%) and the SMR from initial treatment was 6.7 (95% CI, 6.1–7.3). The 5-year OS and SMR among subgroups were shown in Table 1.

A total of 1362 patients were sufficiently followed-up for PFS24 assessment. Of these patients, 1014 (74.4%) were progression-free at 24 months (PFS24 was achieved). The 5-year OS rate after achieving PFS24 was 92.2% (95% CI, 89.6–94.9%; Fig. 1b). The expected 5-year OS rate was 94.3% in the age and sex-matched general Chinese population. The SMR after achieving PFS24 was 1.8 (95% CI, 1.3–2.*3; P* < 0.001). In contrast, the median OS after progression within the first 24 months was only 5.3 months (95% CI, 4.0–6.7). The 5-year OS rate after progression was 19.2% (Fig. 1c) and SMR was 71.4 (95% CI, 62.9–81.0; *P* < 0.001).

### Subgroup analysis of PFS24 and subsequent OS

**Fig. 2:**
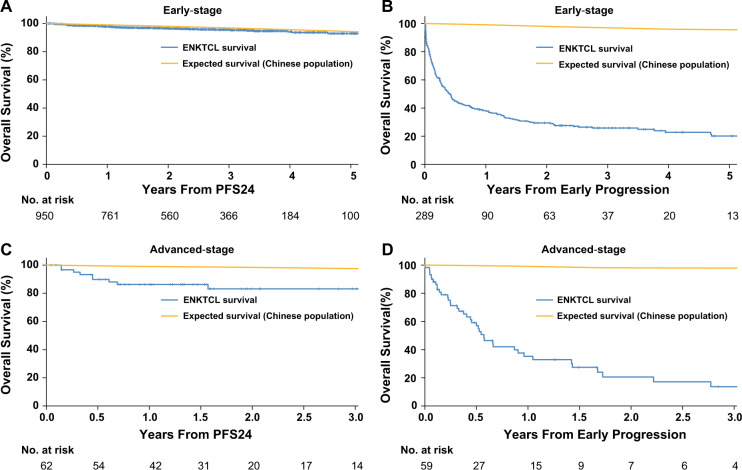
Overall survival (OS) based on 24-month progression-free survival (PFS24) of patients with early-stage and advanced-stage. **a** Subsequent OS of patients who achieved PFS24 after initial treatment vs. expected OS based on age-, sex- and country-matched general population data. **b** Subsequent OS of early-stage patients who did not achieve PFS24 vs. expected OS based on age-, sex-, and country-matched general population data. **c** Subsequent OS of advanced-stage patients who achieved PFS24 vs. expected OS based on age-, sex-, and country-matched general population data. **d** Subsequent OS of advanced-stage patients who did not achieve PFS24 vs. expected OS based on age-, sex-, and country-matched general population data.

In early-stage disease, 950/1239 (76.7%) patients achieved PFS24 and 289/1239 (23.3%) patients did not. The 5-year OS rate after achieving PFS24 was 92.9% (95% CI, 90.2% to 95.6%), almost equivalent to that of the age and sex-matched general population (94.3%, Fig. 2a). The SMR after achieving PFS24 was 1.5 (95% CI, 1.1–2.0; *P* = 0.009). In contrast, the median OS after progression within the first 24 months was only 5.0 months (95% CI, 3.7–6.3). The 5-year OS rate after progression was 20.1% (Fig. 2b) and SMR was 67.3 (95% CI, 58.5- 77.2; *P* < 0.001).

In advanced-stage disease, 123 patients were sufficiently followed-up for PFS24 assessment. PFS24 was achieved for 52.0% patients. The 3-year OS rate after achieving PFS24 was 83.1% (95% CI, 73.4–94.2%), which is lower than that of the age and sex-matched population (97.5%; Fig. 2c). The SMR after achieving PFS24 was 9.6 (95% CI, 5.0–18.4; *P* < 0.001). In contrast, the median OS after progression within the first 24 months was only 6.9 months (95% CI, 4.9–9.1 months), with a 3-year OS rate of 13.7% (Fig. 2d) and SMR of 103.6 (95% CI, 76.0–141.3; *P* < 0.001).

Subsequently, outcomes were examined with regard to other clinical factors and risk groups. Despite a significant difference in OS of patients between treatment subgroups (Table 1), the OS rate of patients after achieving PFS24 were favorable compared with the background population (Table 2). There was little difference in subsequent outcomes after progression regardless of clinical characteristics or risk-stratified grouping (Table 2). PFS24 status was informative for subsequent OS in all patient subgroups and could be used as a prognostic indicator.

**Table 2:** Subsequent outcomes based on achieving or failing PFS24 in subgroups.

	Outcomes from time of achieving PFS24					Outcomes from time of failing PFS24				
	No. of	5-year OS		SMR		No. of	3-year OS		SMR	
Subgroups	patients	% (95%CI)	*P*	(95% CI)	*P*^a^	patients	% (95%CI)	*P*	(95% CI)	*P*^a^
Sex			0.564					0.229		
Male	708	91.2 (87.7–94.9)		1.5 (1.1–2.1)	0.015	242	22.2 (17.0–29.1)		66.9 (57.6–77.8)	<0.001
Female	306	94.6 (91.7–97.6)		3.8 (2.2–6.5)	<0.001	106	29.0 (20.9–40.2)		85.3 (67.5–107.9)	<0.001
Age (years)			0.050					0.856		
≤60	883	93.3 (90.7–96.0)		3.7 (2.7–5.0)	<0.001	305	23.9 (19.1–30.0)		158.2 (138.2–180.9)	<0.001
>60	131	84.8 (75.1–95.9)		0.6 (0.3–1.1)	0.119	43	28.6 (17.4–47.0)		13.9 (9.6–20.1)	<0.001
Primary site			<0.001					0.126		
UADT	982	92.7 (90.1–95.4)		1.6 (1.2–2.1)	0.002	315	24.4 (19.7–30.3)		74.4 (65.2–84.9)	<0.001
Extra-UADT	32	76.2 (60.2–96.5)		9.2 (4.1–20.5)	<0.001	33	21.6 (9.4–49.6)		48.9 (31.2–76.7)	<0.001
Regional lymph nodes			0.030					0.210		
Yes	338	92.0 (88.4–95.7)		3.7 (2.4–5.6)	<0.001	159	20.4 (14.5–28.9)		106.4 (88.5–127.8)	<0.001
No	676	92.7 (89.5–96.0)		1.3 (0.9–1.8)	0.222	189	27.6 (21.3–35.7)		55.1 (46.2–65.6)	<0.001
Distant lymph nodes			0.005					0.944		
Yes	31	85.8 (73.8–99.7)		6.6 (2.5–17.6)	<0.001	30	11.6 (3.4–40.0)		106.2 (69.9–161.3)	<0.001
No	983	92.5 (89.8–95.2)		1.7 (1.2–2.2)	<0.001	318	25.4 (20.6–31.3)		69.1 (60.6–79.0)	<0.001
Primary tumor invasion			0.908					0.006		
Yes	524	93.6 (90.9–96.3)		1.9 (1.3–2.9)	<0.001	220	21.0 (15.7–28.0)		116.0 (99.3–135.5)	<0.001
No	490	91.0 (86.8–95.5)		1.6 (1.1–2.4)	0.015	128	30.2 (22.4–40.6)		40.7 (32.7–50.6)	<0.001
B symptoms			0.472					0.659		
Yes	380	90.8 (85.9–96.0)		2.6 (1.7–3.9)	<0.001	146	24.2 (17.5–33.5)		100.2 (82.2–122.2)	<0.001
No	634	93.1 (90.2–96.0)		1.5 (1.0–2.1)	0.045	202	24.3 (18.5–32.0)		59.6 (50.6–70.3)	<0.001
Elevated LDH			0.053					0.061		
Yes	229	92.2 (88.5–96.1)		3.0 (1.8–4.8)	<0.001	113	16.5 (9.9–27.4)		88.0 (71.1–108.8)	<0.001
No	785	92.3 (89.2–95.5)		1.5 (1.1–2.1)	0.020	235	27.7 (22.1–34.8)		64.7 (55.3–75.8)	<0.001
ECOG score			<0.001					<0.001		
0–1	979	93.3 (91.0–95.6)		1.7 (1.2–2.3)	<0.001	323	26.2 (21.3–32.1)		66.9 (58.6–76.4)	<0.001
≥2	35	56.2 (24.9–100)		3.0 (1.3–6.6)	0.008	25	0		216.9 (142.8–329.4)	<0.001
Ann Arbor stage			<0.001					0.753		
I–II	950	92.9 (90.2–95.6)		1.5 (1.1–2.1)	0.009	289	25.8 (20.9–32.0)		67.3 (58.5–77.2)	<0.001
III–IV	64	83.1 (73.4–94.2)^b^		9.6 (5.0–18.4)	<0.001	59	13.7 (5.9–31.8)		103.6 (76.0–141.3)	<0.001
KPI			0.006					0.492		
Group 1	367	94.1 (90.5–97.9)		1.9 (0.5–1.5)	0.644	83	27.3 (18.9–39.5)		48.0 (37.1–62.1)	<0.001
Group 2	375	90.1 (84.7–95.8)		2.2 (1.4–3.4)	<0.001	124	29.4 (21.6–40.1)		60.6 (48.7–75.5)	<0.001
Group 3	192	95.8 (92.8–98.9)		2.0 (1.0–3.9)	0.056	81	17.4 (11.1–35.4)		131.7 (102.1–170.0)	<0.001
Group 4	80	86.5 (78.5–95.3)		9.3 (4.9–17.9)	<0.001	60	17.0 (8.8–32.7)		111.9 (83.0–150.8)	<0.001
PINK			<0.001					0.928		
Low risk	798	94.5 (91.9–97.2)		2.6 (1.8–3.9)	<0.001	234	26.0 (20.5–32.9)		145.8 (125.1–170.0)	<0.001
Intermediate risk	172	83.9 (75.6–93.0)		1.0 (0.7–1.7)	0.839	69	22.8 (13.7–37.9)		27.5 (20.7–36.6)	<0.001
High risk	44	85.4 (75.2–96.9)		5.0 (2.2–11.1)	<0.001	45	14.3 (5.7 –36.3)		56.2 (39.3–80.4)	<0.001
NRI			0.001					0.186		
Low risk	285	94.5 (90.6–98.7)		2.3 (1.2–4.4)	0.012	48	32.5 (21.2–49.8)		99.2 (70.5–139.6)	<0.001
Intermediate-low risk	314	92.8 (87.8–98.2)		1.0 (0.5–1.8)	0.978	101	27.2 (19.1–38.9)		47.7 (37.4–60.94)	<0.001
Intermediate-high risk	260	90.8 (85.9–96.0)		2.0 (1.3–3.2)	0.003	95	29.2 (20.8–41.1)		77.2 (60.4–98.6)	<0.001
High risk	110	92.5 (87.2–98.1)		2.4 (1.2–5.1)	0.019	69	13.9 (6.4–29.9)		116.6 (88.4–153.9)	<0.001
Very high risk	45	85.1 (74.8–96.8)		2.9 (1.3–6.5)	0.008	35	13.4 (5.1–35.6)^c^		68.6 (47.4–99.4)	<0.001

*PFS24* progression-free survival at 24 months; *SMR* standardized mortality ratio; *OS* overall survival; *UADT* upper aerodigestive tract; *LDH* lactate dehydrogenase; *ECOG* Eastern Cooperative Oncology Group.

^a^Compared with the age- and sex-matched general Chinese population.

^b^3-year OS rate from PFS24.

^c^2-year OS rate from progression.

### Sensitivity analysis of PFS24 vs. other time points

For sensitivity analysis, we examined outcomes according to other landmark time points of PFS, including 12 (PFS12), 24 (PFS24) and 36 (PFS36) months. Although the subsequent 5-year OS rate continued to increase from PFS12 (86.5%) to PFS24 (92.2%), there was little benefit in examining beyond PFS36 (93.0%) and no significant difference between the PFS-based OS and expected OS (Table 3). Furthermore, there was little difference in the 5-year OS rate after progression regardless of the time point chosen (17.1% at 12 months vs. 20.3% at 36 months). Similar results were obtained when considering early-stage and advanced-stage diseases.

**Table 3:** SMR and OS after achieving or failure of PFS at selected time points.

				3 years from time point			5 years from time point		
	No. of	SMR		No. of	Actual 3-y OS^a^	Expected	No. of	Actual 5-y OS^a^	Expected
PFS time point	Patients	(95% CI)	*P*^b^	Patients	% (95% CI)	3-year OS (%)	Patients	% (95% CI)	5-year OS (%)
All stages (I–IV)									
12 months	1274	3.1 (2.6–3.7)	<0.001	594	88.8 (86.9–90.8)	96.9	195	86.5 (84.1–88.9)	94.4
24 months	1014	1.8 (1.3–2.3)	<0.001	380	94.8 (93.2–96.4)	96.9	100	92.2 (89.6–94.9)	94.3
36 months	793	1.0 (0.6–1.6)	0.953	188	97.0 (95.2–98.8)	97.0	32	93.0 (88.7 –97.5)	94.7
Stage I–II									
12 months	1169	2.7 (2.3–3.3)	<0.001	573	90.2 (88.3–92.1)	96.9	190	87.8 (85.4–90.2)	94.4
24 months	950	1.5 (1.1–2.0)	0.009	366	95.5 (94.0–97.1)	96.9	100	92.9 (90.2–95.6)	94.3
36 months	752	1.0 (0.6–1.6)	0.929	183	97.0 (95.2–98.8)	97.0	32	93.0 (88.7–97.6)	94.6
Stage III–IV									
12 months	105	11.4 (7.5–17.4)	<0.001	21	69.7 (59.4–81.7)	97.3	5	69.7 (59.4–81.7)	95.4
24 months	64	9.6 (5.0–18.4)	<0.001	14	83.1 (73.4–94.2)	97.5	–	–	–
36 months	41	2.5 (0.4–18.0)	0.352	5	96.4 (89.8–100)	97.7	–	–	–
All stage (I–IV)									
12 months	246	65.8 (56.8–76.2)	<0.001	27	22.9 (17.9–29.5)	96.4	8	17.1 (11.6–25.2)	94.8
24 months	348	71.4 (62.9–81.1)	<0.001	40	24.3 (19.8–30.0)	97.1	14	19.2 (14.3–25.9)	95.8
36 months	388	55.0 (48.7–62.0)	<0.001	41	25.4 (20.9–30.8)	96.4	14	20.3 (15.3–26.8)	93.2
Stage I–II									
12 months	201	62.7 (53.3–73.7)	<0.001	25	23.9 (18.4–31.1)	96.3	7	17.4 (11.5–26.4)	94.6
24 months	289	67.3 (58.5–77.2)	<0.001	37	25.8 (20.9–32.0)	97.0	13	20.1 (14.7–27.4)	95.6
36 months	325	50.4 (44.2–57.6)	<0.001	38	26.9 (22.1–32.9)	96.3	13	21.2 (15.9–28.4)	92.8
Stage III–IV									
12 months	45	85.9 (60.4–122.2)	<0.001	2	14.5 (5.6–37.4)	97.4	–	–	–
24 months	59	103.6 (76.0–141.3)	<0.001	3	13.7 (5.9–31.8)	97.9	–	–	–
36 months	63	103.4 (76.4–140.0)	<0.001	3	13.8 (6.0–31.9)	97.9	–	–	–

*SMR* standardized mortality ratio, *OS* overall survival, *PFS* progression-free survival.

^a^Actual 3- or 5-year OS was defined as subsequent OS of patients who achieved or failed to achieve PFS at 12, 24 or 36 months after initial treatment based on patients with ENKTCL vs. expected 3- or 5-year OS based on age-, sex- and country-matched general population data.

^b^Compared with the age- and sex-matched general Chinese population.

## Discussion

This is the first study to evaluate PFS-based endpoints among patients with ENKTCL, primarily treated with non-ANT-based chemotherapy and frontline radiotherapy. Using a large multicenter cohort of patient data collected from the CLCG database, we verified that PFS24 acts as a prognostic indicator of subsequent survival under current treatment strategies. Patients who were progression-free within 24 months after initial treatment resulted in favorable long-term outcomes, with OS times not distinguishable from that of age-, sex- and country-matched populations. Conversely, patients who experienced disease progression within 24 months had poor prognoses. This finding suggests PFS24 as a dichotomous variable, providing a clear benchmark for evaluating the success of initial treatment and for designing clinical trials for ENKTCL.

The introduction of asparaginase- or platinum-based chemotherapy regimens and early implementation of high-dose E-ISRT for localized disease have been the most important advances in the treatment of ENKTCL [11–21, 31]. Although phase I/II trials or retrospective studies have reported improved survival with the use of non-ANT-based chemotherapy, the optimal chemotherapy regimens remain to be identified by prospective trials. Early efficacy endpoints such as PFS and EFS are required to reduce the duration of evaluation of treatment regimens, to deliver optimal treatment strategies to the clinic sooner, and so that ineffective regimens can be abandoned without prolonged evaluation [32]. However, due to the rarity of ENKTCL and the heterogeneity of treatments used, limited patient numbers make formal surrogate endpoint analysis difficult. Normalization to the survival of a matched general population is important in rare diseases such as PTCL and ENKTCL [29]. In the present study, PFS24 is identified as a dichotomous variable that may allow individualized prognosis prediction for patients with ENKTCL, as well as inform patient counseling, clinical decision making and prospective clinical trial design.

In previous studies, we have demonstrated that improved locoregional control is associated with prolonged PFS and OS in early-stage ENKTCL [33], and that the survival probability increased and the hazards of failure decreased in a risk-dependent manner [34]. In the present study, we demonstrate that the estimated risk of disease progression and mortality was initially high, but decreased dramatically within the first 24 months after current standard treatment for ENKTCL (Fig. 1a). Given that the vast majority of ENKTCL-related events occur within the first 24 months, patients achieving PFS24 are highly likely to have a near-normal life expectancy, with actual and expected 5-year OS rates of 92.2% and 94.3%. Sensitivity analysis, examining time points at 12, 24, and 36 months, indicated that there was little OS improvement at PFS36, whereas the PFS12 endpoint did not dichotomize the two groups as effectively as PFS24, regardless of stage. In consistence with the observations of the current study, other studies have reported PFS24 to be an important endpoint for DLBCL and PTCL, associated with subsequent long-term outcomes [28, 29]. Regarding ENKTCL patients who failed to achieve PFS24, the median OS after progression was only 5.3 months, indicating that few patients who suffer early progression can be successfully treated. As observed by other studies [35, 36], ENKTCL patients who suffer disease progression after initial treatment had extremely poor prognoses, regardless of stage or risk group. In a recent study of 179 patients with ENKTCL who suffered disease relapse or progression after initial non-ANT-based chemotherapy, the median OS was only 6.4 months [35]. Similarly, for patients who failed to achieve PFS24 or EFS24, the subsequent median OS was 7.2 months after ANT-based immunochemotherapy for DLBCL and 4.9 months after ANT-based chemotherapy for PTCL [28, 29]. These findings indicate that PFS24 could be safely incorporated into routine clinical practice without detrimental effects on long-term disease control or survival outcomes in aggressive lymphomas under current standard treatments. PFS24 should be further validated in the context of randomized controlled trials as a potential early efficacy endpoint for patients with ENKTCL. Furthermore, there is an urgent requirement to identify patients at high risk of early progression who would benefit from innovative treatment strategies. Following the achievement of PFS24, research for ENKTCL survivors should focus on less-intensive surveillance, long-term treatment toxicity, quality of life, and other outcomes [34, 37, 38].

Previous studies, mainly using the randomized controlled trial (RCT) or real-world data, have demonstrated that the surrogate endpoints of EFS and PFS are strongly related to OS at both trial- and individual-level in different lymphoma subtypes [26–29, 32, 39–41]. Consistent with the present finding, the subsequent survival of patients with DLBCL or PTCL who achieved EFS24 or PFS24 is almost equal to that of the age-, sex-, and country-matched general population [26–29, 32]. Furthermore, we have confirmed the association of PFS24 with OS in trial- and treatment arm-level in RCTs on DLBCL patients treated with immunochemotherapy; its association has been externally validated using the literature-based data from high-quality phase II and retrospective studies [40]. Similarly, in patients with follicular lymphoma and marginal zone lymphomas [39, 41], early progression of disease within 24 months after initial treatment stratified subsequent OS and identifies a high-risk population. Consistent with the present study, these findings highlight the important role of PFS or EFS as early efficacy endpoint in designing prospective trials. The further work is needed to determine the prognostic factors for PFS24 in patients with ENKTCL.

The limitations of this study are as follows: first, the impact of salvage treatment on survival outcome after progression was not assessed, as it is beyond the scope of this study. Originally, our dataset was designed to collect only baseline characteristics, initial treatments, and events during follow-up. Second, these findings were mostly based on patients with early-stage disease, primarily treated with non-ANT-based regimens and radiotherapy. Extrapolation of these results when considering patients who underwent other treatments would be speculative. Third, despite the wide enrollment of ENKTCL patients primarily treated with non-ANT-based chemotherapy and upfront radiotherapy from a real-world CLCG database, more work is required to externally validate the correlation between PFS24 and OS using independent data from other large database, particularly from nonendemic areas. Furthermore, the retrospective design of this study does not allow for assessment of EFS as endpoint. The evaluation of EFS24 vs. PFS24 may be explored in a future study using CLCG data. The patients included in this study represent a young population with few comorbidities; longer follow-up and comparison to other collaboration data will give more insight into the importance of these endpoints.

In conclusion, assessment of PFS24 stratifies subsequent outcomes in patients with ENKTCL. Patients who achieve PFS24 have favorable subsequent survival regardless of stage and risk group. However, patients who suffer early progression have extremely poor prognoses. These results support the use of PFS24 as an efficacy endpoint, and should be considered in future studies evaluating novel therapies and biomarkers for risk stratification.
